# Genome Evolution of a Symbiont Population for Pathogen Defense in Honeybees

**DOI:** 10.1093/gbe/evac153

**Published:** 2022-10-20

**Authors:** Karl Dyrhage, Andrea Garcia-Montaner, Daniel Tamarit, Christian Seeger, Kristina Näslund, Tobias C Olofsson, Alejandra Vasquez, Matthew T Webster, Siv G E Andersson

**Affiliations:** Department of Molecular Evolution, Cell and Molecular Biology, Biomedical Centre, Science for Life Laboratory, Uppsala University, Uppsala, Sweden; Department of Molecular Evolution, Cell and Molecular Biology, Biomedical Centre, Science for Life Laboratory, Uppsala University, Uppsala, Sweden; Department of Molecular Evolution, Cell and Molecular Biology, Biomedical Centre, Science for Life Laboratory, Uppsala University, Uppsala, Sweden; Department of Molecular Evolution, Cell and Molecular Biology, Biomedical Centre, Science for Life Laboratory, Uppsala University, Uppsala, Sweden; Department of Molecular Evolution, Cell and Molecular Biology, Biomedical Centre, Science for Life Laboratory, Uppsala University, Uppsala, Sweden; Department of Laboratory Medicine, Lund University, Lund, Sweden; Department of Laboratory Medicine, Lund University, Lund, Sweden; Department of Medical Biochemistry and Microbiology, Science for Life Laboratory, Uppsala University, Uppsala, Sweden; Department of Molecular Evolution, Cell and Molecular Biology, Biomedical Centre, Science for Life Laboratory, Uppsala University, Uppsala, Sweden

**Keywords:** defensive symbionts, evolution, *Apilactobacillus kunkeei*, plasmids, mobile elements, transposons

## Abstract

The honeybee gut microbiome is thought to be important for bee health, but the role of the individual members is poorly understood. Here, we present closed genomes and associated mobilomes of 102 *Apilactobacillus kunkeei* isolates obtained from the honey crop (foregut) of honeybees sampled from beehives in Helsingborg in the south of Sweden and from the islands Gotland and Åland in the Baltic Sea. Each beehive contained a unique composition of isolates and repeated sampling of similar isolates from two beehives in Helsingborg suggests that the bacterial community is stably maintained across bee generations during the summer months. The sampled bacterial population contained an open pan-genome structure with a high genomic density of transposons. A subset of strains affiliated with phylogroup A inhibited growth of the bee pathogen *Melissococcus plutonius*, all of which contained a 19.5 kb plasmid for the synthesis of the antimicrobial compound kunkecin A, while a subset of phylogroups B and C strains contained a 32.9 kb plasmid for the synthesis of a putative polyketide antibiotic. This study suggests that the mobile gene pool of *A. kunkeei* plays a key role in pathogen defense in honeybees, providing new insights into the evolutionary dynamics of defensive symbiont populations.

**Significance**

The contribution of the beehive microbiota to the health of honeybees is poorly understood. The bacterium *Apilactobacillus kunkeei* is highly abundant in the honey crop and the honeybee food products. By comparing the genomes and mobilomes of more than 100 novel *A. kunkeei* isolates, we found that some strains contained plasmids for molecular defense systems. Our in vitro studies confirmed that strains that contained the plasmid-encoded biosynthetic gene cluster for enzymes involved in the synthesis of kunkecin A inhibited growth of the bee pathogen *Melisococcus plutonius*. This plasmid was stably maintained in strains obtained from one of four beehives throughout the summer months. We propose that *A. kunkeei* is a defensive symbiont of honeybees and that its mobilome provides a dynamic protection system against *M. plutonius*.

## Introduction

Symbiotic relationships between bacteria and eukaryotes are common in nature, affecting the physiology, development, behavior, and growth habitat of the host. Many insects house nutritional symbionts, which serve as small bacterial factories for the production of amino acids, vitamins, or other compounds lacking in the host diet (reviewed in Dale and Moran 2006; Toft and Andersson 2010; Bennet and Moran 2015). Most obligate nutritional symbionts have undergone reductive genome evolution and massive gene loss, whereas genes that serve host–beneficial functions have evolved under strong selective pressures.

Defensive symbionts, which provide protection against parasites and pathogens, are also widespread in insects (reviewed in White and Torres 2009; Clay 2014; Ford and King 2016; King 2018; Vorburger and Perlman 2018). These bacteria offer protection from infectious diseases by secreting antimicrobial compounds and/or by stimulating the host immune system (reviewed in Van Arnam et al. 2018). The symbiotic relationships are more complex and dynamic than those of obligate nutritional symbionts, and the genomes of defensive symbionts are large. However, one-third of the protein coding genes in the 7 Mb genome of the vertically inherited actinobacterial symbionts of solitary beewolf wasps contain frameshift mutations, suggesting that it is an early stage of genome reduction (Nechitaylo et al. 2021). In *Lagria* beetles, one strain of *Burkholderia gladioli* has a genome of about 2.3 Mb, whereas other coexisting strains of the same species have genomes of 8.5 Mb (Floréz et al. 2018). Advanced stages of genome reduction are very rare but has been observed in *Candidatus* Profftelia armature which lives inside bacteriocytes in its psyllid host and has a drastically reduced genome of only 465 kb, 15% of which contain genes for the synthesis of a polyketide toxin (Nakabachi et al. 2013).

The molecular defense systems may be encoded by horizontally acquired chromosomal genes, as in *Candidatus* Profftelia armature (Nakabachi et al. 2013), or associated with mobile elements, such as plasmids in *Pseudonocardia* symbionts of fungus-growing ants (Gerardo and Parker 2014; Van Arnam et al. 2015), or phages, as in the *Hamiltonella* symbionts defending aphids against parasitoid wasps (Degnan and Moran 2008; Oliver et al. 2009). Yet, very little is still known about the mobility of host-selected bacterial defense systems, and the evolutionary dynamics of defensive symbiont populations have rarely been explored beyond comparisons of 16S rRNA and genome sequence identities.

Honeybees provide an attractive model system for evolutionary studies of host-associated microbial communities and their influence on host health (Engel et al. 2016; Kwong and Moran 2016; Zheng et al. 2018; Nowak et al. 2021). Honeybees are social organisms who share their food sources (nectar and pollen) and nurse the next generation of bees in a collaborative manner. They establish colonies comprising 30,000–80,000 adult workers. As in human societies, their social life style makes the colonies vulnerable to infections by viruses, parasites and bacterial pathogens. The most common infectious disease agents of honeybees are microsporidians and other fungi, RNA viruses transmitted by the *Varroa* mite, and bacteria such as *Paenibacillus larvae* and *Melissococcus plutonius*, which causes larval foulbrood disease (reviewed in Funfhaus et al. 2018; Li et al. 2018a, 2018b). Altogether, these infections pose severe threats to honeybee colonies worldwide and are of great economic concern for farmers and the global commercial honeybee industry.

Honeybees use a variety of defense mechanisms to tackle the threats from infectious diseases, including changes in social behavior, establishment of physical barriers in the gut and induction of apoptosis and immune response systems (Doublet et al. 2017; Li et al. 2018a, 2018b). Some protection against infectious diseases may also be obtained from the gut microbiome, which consists of about 10^8^ to 10^9^ bacterial cells per worker bee. The core bee gut microbiome consists of only a few bacterial genera, including several species of *Snodgrasella*, *Gilliamella*, *Lactobacillus* and *Bifidobacterium* (Martinson et al. 2011; Engel et al. 2012, 2014; Moran et al. 2012; Sabree et al. 2012; Jones et al. 2017; Ellegaard and Engel 2019). A few additional, but less prevalent species have also been identified, such as *Frischella perrara*, which solely infects the pylorus (Engel et al. 2013). The establishment of the core lineages in the gut coincided with the diversification of eusocial corbiculate bees from solitary bees (Kwong et al. 2017a, 2017b).

The composition of the gut microbiome in worker bees has been shown to be influenced by infections of *M. plutonius* (Erban et al. 2017), as well as by the administration of the honeybee gut bacterium *Snodgrassella alvi* and the protozoan parasite *Lotmaria passim* (Schwarz et al. 2016). Comparative studies of bees with and without a microbiota provided support for the hypothesis that the gut microbiome exerts an effect on the host immune system (Kwong et al. 2017a, 2017b). *Frischella perrara* in particular has a strong effect on the immune system, possibly indicating that it is a pathogen rather than a mutualist (Emery et al. 2017). However, the mechanisms involved and the impact of the individual members of the gut microbiome on the health of individual worker bees are still poorly understood (discussed in Engel et al. 2016).

*Apilactobacillus kunkeei* (formerly named *Lactobacillus kunkeei*) is a fructophilic lactic acid bacterium (Endo et al. 2012) and the dominant bacterial species in the honey crop (Vasquez et al. 2012), which is the first stomach (or sac) in the gut used for storage and transport of nectar to the hive. *Apilactobacillus kunkeei* has also been found on flowers as well as in the honeybee food products, such as fresh honey, bee bread, and royal jelly (Olofsson and Vasquez 2008; Vasquez and Olofsson 2009; Anderson et al. 2013; Endo and Salminen 2013; Tamarit et al. 2015; Anderson and Ricigliano 2017). Because of its broad prevalence in the food sources of honeybees, *A. kunkeei* has often been viewed as an environmental hive bacterium rather than a core gut symbiont (Anderson et al. 2013; Anderson and Ricigliano 2017; Kwong et al. 2017a, 2017b). Yet increasingly, it is recognized that *A. kunkeei* may be a key player in the defense against pathogens and parasites in honeybee colonies.

For example, it has been shown that *A. kunkeei* can inhibit the growth of the bacterium *P. larvae* (Forsgren et al. 2010; Butler et al. 2013; Kacaniova et al. 2020), the microsporidian *Nosema ceranae* (Arredondo et al. 2018), as well as the fungus *Ascosphaera apis* (Iorizzo et al. 2020). Growth inhibition of *P. larvae* was also observed when cell-free supernatants of *A. kunkeei* were used, suggesting that the antimicrobial effects were caused by secreted metabolites and/or proteins (Lamei et al. 2019). Recently, a strain of *A. kunkeei* FF30-6 that exhibits antibacterial activity (Endo and Salminen 2013) was found to contain a plasmid (pKUNF330) that codes for enzymes involved in the synthesis of a novel antimicrobial compound, named kunkecin A (Zendo et al. 2020). This metabolite has a narrow antimicrobial spectrum with high activity against *M. plutonius* (Zendo et al. 2020), suggesting that *A. kunkeei* strains hosting this plasmid serve a role as defensive symbionts of honeybees.

The few genomes sequenced until now, only one of which is closed (Asenjo et al. 2016), indicate extensive gene content diversity among strains despite near 16S rRNA identity (Tamarit et al. 2015). Notably, the *A. kunkeei* genome is functionally structured such that genes encoding metabolic functions are located near the origin of replication, whereas genes for basic information processes are located around the terminus (Tamarit et al. 2015). Despite the many advances that the genomic studies have offered, it is not known if the plasmid for kunkecin A biosynthesis is widespread in the *A. kunkeei* population and if additional plasmids with defensive functions are circulating in the honeybee growth niche.

In this study, we compare the closed genomes and the associated mobilomes of 102 new *A. kunkeei* isolates along with re-sequenced genomes of *A. kunkeei* Fhon2 and *Apilactobacillus apinorum* Fhon13. The novel strains were sampled from the honey crop of honeybees in the south of Sweden (Helsingborg), as well as from bees at two geographically isolated islands in the Baltic Sea (Åland and Gotland). The Gotland bees are a mixture of European ancestries caused by importation from other parts of Europe and fairly typical of managed honeybees all over Sweden (Wallberg et al. 2014). However, whereas the Gotland bees belong to a bee population that lives in contact with the *Varroa* mite and has developed a natural resistance to it, the bees from Åland come from a *Varroa* mite-free area (Fries et al. 2006; Lattorff et al. 2015; Thaduri et al. 2018). Thus, the sampled honeybee populations have evolved under distinct ecological settings. We present an overview of the mobile gene pool in the *A. kunkeei* population and discuss its role in food preservation and health in honeybee colonies.

## Results

### Genome Sequencing of 102 Novel *A. kunkeei* Isolates

**Fig. 1.**
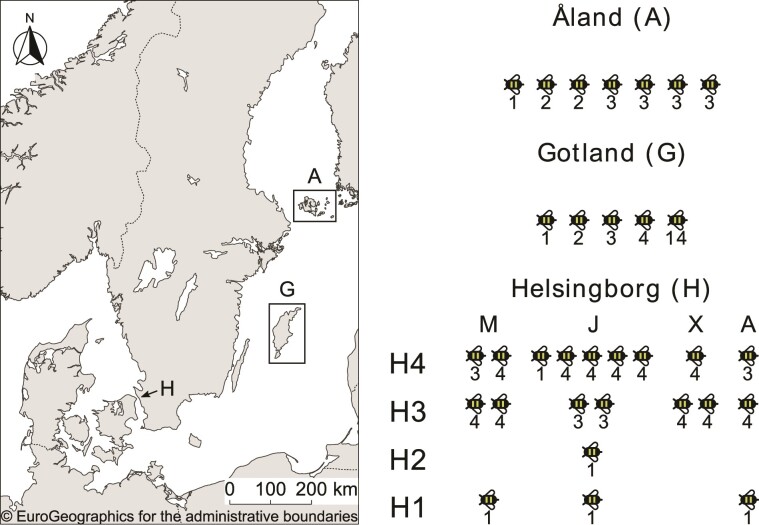
Origin of samples and *A. kunkeei* strain code nomenclature. Schematic illustration of the origins of the cultivated *A. kunkeei* strains obtained from honeybee hives located on the islands Åland and Gotland in the Baltic Sea and from hives sampled between May and August in Helsingborg, Sweden. Numbers below honeybees represent the number of *A. kunkeei* strains sampled from each bee. The bacterial strains from Åland and Gotland were named indicating the island (e.g., G0104 or A0901), the host bee (e.g., G0104) and the bacterial isolate (e.g., G0104). The bacterial strains obtained from Helsingborg were named following an alphanumerical system indicating the site (e.g., H3B2-04M), the hive number (e.g., H3B2-04M), the host bee (e.g., H3B2-04M), the bacterial isolate (e.g., H3B2-04M) and the month in which the sample was collected (e.g., H3B2-04M). Abbreviations for months; M = May, J = June, X = July, A = August.

Bacterial colonies were isolated from the honey crop of honeybees sampled from four beehives (fig. 1). In total, 102 novel bacterial isolates were obtained, of which 61 isolates were from Helsingborg, 17 from Åland and 24 from Gotland. At all sites, 1–7 bees were collected per beehive and sampling time point, of which 1–14 bacterial colonies per bee were isolated (supplementary table S1, Supplementary Material online). Samples from beehives in Helsingborg were taken as a time series from May to August. The generation times of the new *A. kunkeei* isolates in fMRS media ranged from 34 to 61 min, with an average of 48.3 min (calculated from the mean of the mean growth rate for each isolate) (supplementary tables S2 and S3, Supplementary Material online).

The genomes of the novel isolates as well as the re-grown strains *A. kunkeei* Fhon2 and *A. apinorum* Fhon13, altogether 104 genomes, were sequenced and assembled into single contigs with sizes ranging from 1.49 to 1.64 Mb (mean 1.57 Mb) (supplementary table S3, Supplementary Material online). The genomes contained 5 rRNA operons, 65–72 tRNA genes and 1,345–1,504 (mean 1,430) protein coding genes (supplementary table S3, Supplementary Material online). Plasmids and phage–plasmids of 20–40 kb were identified in 40 isolates (supplementary table S3, Supplementary Material online). The ratios of sequence read coverage over these elements compared with the chromosome were on the average equal to or lower than one for each type of plasmid and phage-plasmid (supplementary fig. S1, Supplementary Material online).

### The Diversity of the Sampled Bacterial Isolates is Specific for Each Beehive

**Fig. 2.**
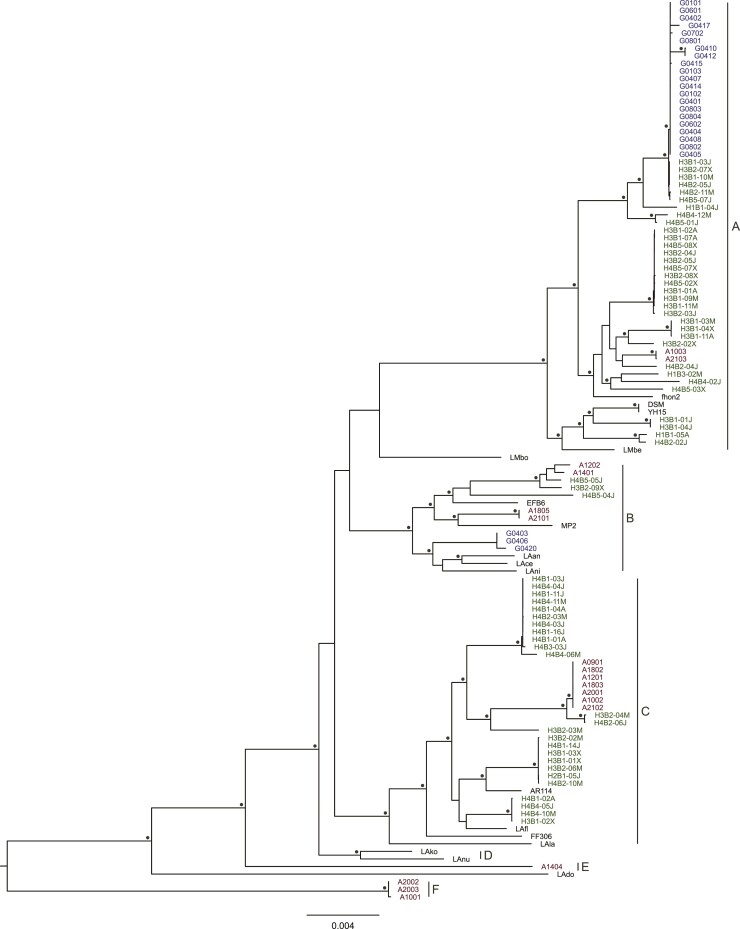
Phylogenetic representation of *A. kunkeei* strains. The strains have been color-coded to represent the site from which they were obtained; blue = Gotland; green = Helsingborg; brown = Åland; black = previously published genomes (Djukic et al. 2015; Porcellato et al. 2015; Sun et al. 2015; Tamarit et al. 2015; Asenjo et al. 2016). Letters A–F indicate phylogroup designations. The phylogeny was obtained using IQ-Tree with the LG + F + R5 model based on 682 single-copy orthologous proteins in 119 *A. kunkeei* strains and *A. apinorum* Fhon13. The tree is displayed such that branch lengths are proportional to substitution frequencies. The statistical support for the branching pattern was estimated from 1,000 ultrafast bootstraps and SH-like pseudoreplicates. Nodes supported by 100% by both methods are highlighted with filled circles. For ease of visualization, the branch to *A. apinorum* Fhon13 is not displayed. The full tree, including *A. apinorum* Fhon13 and the statistical support values for all nodes are shown in supplementary figure S2, Supplementary Material online.

Pairwise 16S rRNA sequence identities were calculated for each of the 102 novel isolates in comparisons to *A. kunkeei* Fhon2 and *A. apinorum* Fhon13. The values ranged from 99.4% to 100% to *A. kunkeei* strain Fhon2 and from 98.4% to 99.2% to *A. apinorum* strain Fhon13 (supplementary table S3, Supplementary Material online). Moreover, a maximum likelihood phylogenetic tree (fig. 2; supplementary fig. S2, Supplementary Material online) based on a concatenated alignment of 682 proteins (supplementary table S4, Supplementary Material online) showed that all isolates obtained in this study clustered with the previously described *A. kunkeei* strains to the exclusion of *A. apinorum* Fhon13.

The tree topology indicated that 95% of the novel *A. kunkeei* isolates belonged to three main clades, corresponding to the previously suggested phylogroups A, B, and C (fig. 2; supplementary fig. S2, Supplementary Material online). The remaining four isolates formed two deeply diverging lineages, here named phylogroups E and F. The average nucleotide identity (ANI) values for strain comparisons between phylogroups A, B and C were about 95–96%, while pairwise comparisons of these strains to those classified into phylogroups E and F were in the range of 90–93% (supplementary table S5, Supplementary Material online). Thus, based on the 16S rRNA analysis alone, all novel isolates should be classified as strains of the *A. kunkeei* species, while the ANI values suggest that the four isolates from Åland in phylogroups E and F should be considered as belonging to a different species. For now, we have named them as novel strains of the *A. kunkeei* species, while awaiting taxonomic evaluation.

The large majority of isolates obtained from honeybees from the island Gotland belonged to phylogroup A, while a few represented a rare variant in phylogroup B obtained from a single bee that was sampled more deeply. Samples from the island Åland contributed isolates from most phylogroups and the sampled bees contained mostly isolates from two and sometimes even three different phylogroups, indicative of a more diverse strain co-occurrence profile. The *A. kunkeei* isolates obtained from Helsingborg were evenly distributed between the A and C phylogroups in addition to a few isolates affiliated with phylogroup B. However, there was a bias such that the A-group strains were more abundant in isolates cultivated from beehive 3, whereas the C-group strains dominated among isolates obtained from beehive 4. The community profiles of the isolates from Helsingborg were largely specific for each of the two beehives and remarkably stable during the summer months (May–August).

### The Pan-Genome Structure is Open

**Fig. 3.**
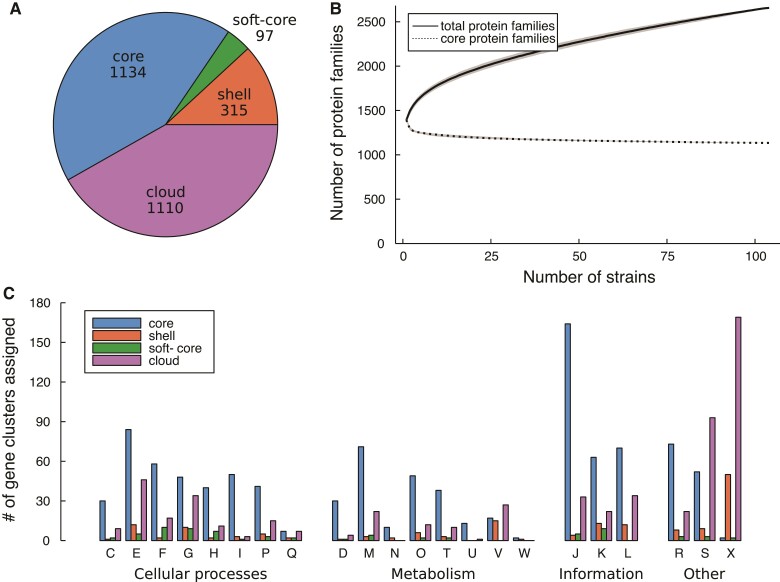
Gene contents in the *A. kunkeei* population. (*A*) The pie chart shows the division of 2,656 protein families in the *A. kunkeei* population into core families and three categories of accessory protein families (shell, soft-core and cloud). The families were obtained from chromosomally encoded proteins in 104 *A. kunkeei* isolates. (*B*) The number of protein families plotted against the number of isolates for the conserved core families and the total set of families. Isolates were sampled randomly 10,000 times without replacement. Full and dashed lines show mean number of protein families, and the gray areas show upper and lower bounds. (*C*) Functional analyses of core and variably present protein families. The number of core and variable protein families is shown as a function of COG category for the proteome of the *A. kunkeei* population. One-letter abbreviations and full names of COG categories: Amino acid transport and metabolism (*E*), carbohydrate transport and metabolism (*G*), cell cycle control, cell division, chromosome partitioning (*D*), cell motility (*N*), cell wall/membrane/envelope biogenesis (*M*), chromatin structure and dynamics (*B*), coenzyme transport and metabolism (*H*), defense mechanisms (*V*), energy production and conversion (*C*), extracellular structures (*W*), function unknown (*S*), general function prediction only (*R*), inorganic ion transport and metabolism (*P*), intracellular trafficking, secretion, and vesicular transport (*U*), lipid transport and metabolism (*I*), mobilome: prophages, transposons (*X*), nucleotide transport and metabolism (*F*), posttranslational modification, protein turnover, chaperones (*O*), RNA processing and modification (*A*), replication, recombination and repair (*L*), secondary metabolites biosynthesis, transport and catabolism (*Q*), signal transduction mechanisms (*T*), transcription (*K*), translation, ribosomal structure and biogenesis (*J*).

The pan-genome structure of the *A. kunkeei* population was examined based on 2,656 protein families (supplementary table S4, Supplementary Material online) that contained proteins encoded by chromosomal genes in the 104 isolates with closed genomes (fig. 3*A*). In addition to the 1,134 protein families shared by all isolates, the “soft-core” proteome (shared by 95–99% of the strains) consisted of 97 families, the “shell” proteome (shared by 15–95% of the genomes) contained 315 families, and the “cloud” proteome (shared by 0–15% of the genomes) included 1,110 families, of which 620 were specific for a single strain. The cumulative number of families continued to increase with the addition of new isolates, showing no sign of flattening (fig. 3*B*). The core proteins encoded by genes present in all genomes were mostly involved in information processes and metabolic systems, while defense systems and mobile genetic elements were over-represented among the shell and cloud proteins (fig. 3*C*; supplementary table S4, Supplementary Material online). Thus, the sampled *A. kunkeei* community retained an open pan-genome structure despite being represented by over 100 genomes, which suggests high ecological plasticity in the species.

### The Genomes Contain Prophages and a High Density of Transposons

**Fig. 4.**
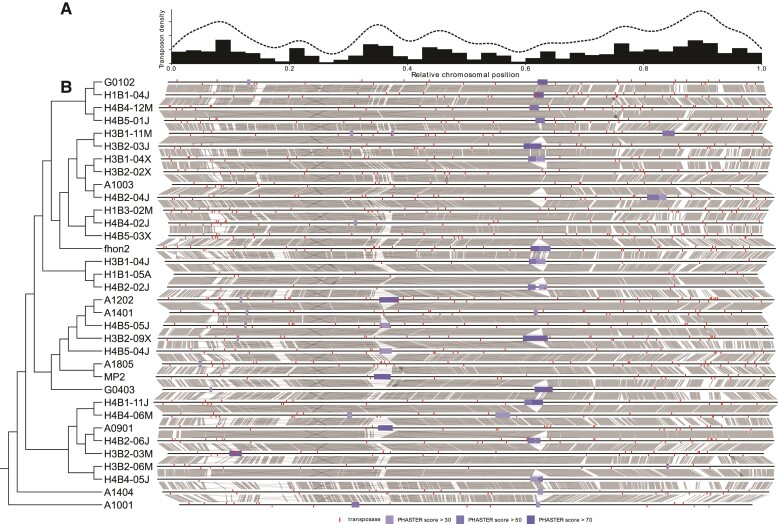
Distribution of transposons and prophages. (*A*) Density plot representing the distribution of transposase genes across the genome in 34 representative strains of *A. kunkeei.* (*B*) The location of transposases and prophages in genomes from 34 isolates, including only one representative genome among those that are more than 99.9% identical. The tree topology is schematic and taken from figure 2. Vertical red lines above or below chromosome lines indicate transposase genes located in the Watson or Crick strands, respectively. Blue boxes on the chromosomes represent prophage genes predicted by PHASTER (score more than 30). Gray, connecting lines represent best-reciprocal Blastn hits with *e*-values lower than 1*e*−5. An equivalent plot with all complete genomes included in this study is shown in supplementary figure S3, Supplementary Material online.

Most genomes contained a prophage of 35–50 kb located at either of two sites around the terminus of replication (between positions ∼600,000 and ∼1,000,000, respectively) (fig. 4; supplementary fig. S3, Supplementary Material online). Furthermore, all genomes contained a phage defense island, which encoded either a CRISPR-Cas system or a restriction-modification system, or a prophage as in the MP2 genome (supplementary fig. S4, Supplementary Material online).

On average, we identified 52 transposase genes per genome (ranging from 2–90 per genome), many of which were found in pairs and belonged to the IS3 family (subgroup IS150) (fig. 4; supplementary fig. S3, Supplementary Material online). A plot of the average number of transposases along 34 reference genomes (selected to represent groups of genomes with ANI values of 99.9%) revealed multiple transposon-dense areas (fig. 4*A*). In the chromosomal half that flanks the origin of replication, these were often associated with chromosomal segments that were variable in gene content (fig. 4*B*; supplementary fig. S3, Supplementary Material online), suggesting that much of the genomic variability in the *A. kunkeei* population is due to transposon-mediated activities. Indeed, we identified genomic heterogeneity within some of the isolates in the form of transposon-containing contigs that were homologous to chromosomal segments. One such contig in strain H3B2-03M contained genes that were homologous to genes for enzymes involved in exopolysaccharide synthesis and biofilm formation located in a chromosomal segment of 30.3 kb flanked by transposons. This strain segregated upon repeated culturing and plating into colonies with different growth kinetics (supplementary fig. S5 and supplementary table S2, Supplementary Material online). Resequencing showed that the extrachromosomal contig was retained in one colony, but lost in two colonies of which one had also lost the 30.3 kb chromosomal segment (supplementary table S3, Supplementary Material online).

### Key Pathogen Defense Systems are Encoded by Plasmids

**Fig. 5.**
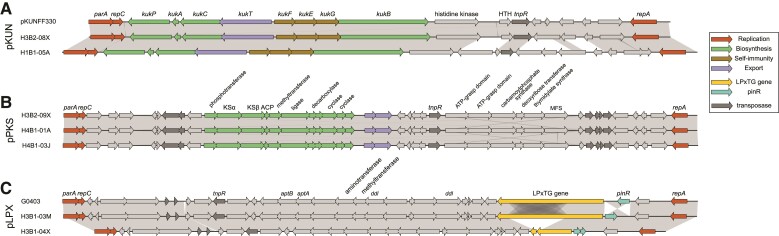
Genes located on plasmids in *A. kunkeei* strains. Synteny plots for (*A*) the 19.5 kb pKUN plasmid carrying genes for the biosynthesis of kunkecin A, (*B*) the 33.5 kb pPKS plasmid carrying genes for the biosynthesis of putative novel polyketide antibiotic, and (*C*) the 33 kb plasmid carrying a gene for the cell surface protein with the LPxTG motif. Detailed information about the annotation of plasmid genes is provided in supplementary table S6, Supplementary Material online.

We identified three plasmids and two phage–plasmids in the 20–40 kb size range that contained a gene for the replication protein RepA located next to a gene for the plasmid partitioning protein ParA (fig. 5; supplementary table S6, Supplementary Material online). The three plasmids contained genes for accessory mobile elements, such as IS3 family transposases and the Tn3 family resolvase, as well as genes for adhesins or enzymes putatively involved in the synthesis of antibiotics, as detailed below. The two phage–plasmids contained phage genes for the production of phage particles, host cell lysis and infection in addition to the *repA* and *parA* genes (supplementary table S6, Supplementary Material online). The phage proteins showed homology to phage protein families pLP39 and p48, respectively, which have been identified previously in other *Lactobacillus* species (Pfeifer et al. 2021). In addition, we identified smaller replicons in the 7–10 kb size range, some of which contained a gene for the rolling-circle initiator protein RepB as well as genes coding for macrolide export and ESX secretion systems or DNA methylases and DNA restriction enzymes.

The 19.5 kb pKUN plasmid contained a cluster of eight consecutive genes for the synthesis of kunkecin A, a nisin-like antibiotic (fig. 5*A*; supplementary table S6, Supplementary Material online). The cluster included genes for the kunkecin A precursor peptide, the leader peptidase, proteins involved in the modification and transport of the antibiotic and proteins conferring self-immunity, as in the plasmid described in (Zendo et al. 2020). Downstream of this gene cluster, we identified a gene coding for a protein with a histidine kinase domain and a gene for a protein with a helix-turn-helix motif. We suggest that these two genes code for homologs to the NisR/NisK two-component regulatory system, which were previously thought to be missing from the kunkecin A biosynthetic gene cluster (Zendo et al. 2020).

The 32.9 kb plasmid was named pPKS because it contained a cluster of genes for polyketide synthesis (fig. 5*B*; supplementary table S6, Supplementary Material online). This plasmid contained three genes for the beta-ketoacyl synthetase subunits KSα and Ksβ and the acyl carrier protein, which constitute the minimal polyketide synthase complex. The cluster also contained genes for enzymes involved in methyl transfer, decarboxylation and cyclization of polyketide antibiotics. Additionally, the plasmid contained three genes coding for proteins with high sequence similarity to ATP-grasp domain proteins in *Streptococcus* and *Lactobacillus* species (1e^−95^).

**Fig. 6.**
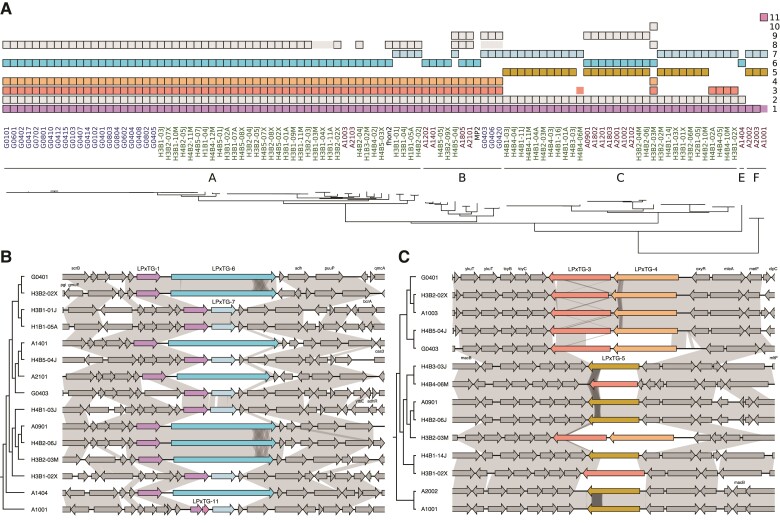
Phyletic distribution patterns and gene order structures for *A. kunkeei* proteins with LPxTG-domains. (*A*) Phyletic distribution patterns of proteins containing the LPxTG domain as predicted by InterProScan. The phylogeny has been taken from figure 2 and the colors of the isolates correspond to the phylogroups. Protein families containing surface proteins with LPxTG motifs, indicated by numbers 1–11 on the right side and arbitrarily named LPxTG-1 to LPxTG-11, are color-coded to represent different orthogroups. Proteins clustered into these orthogroups but without an identified LPxTG domain are shown as boxes without a black border. Gene synteny is shown for a few representative isolates for protein families classified as (*B*) LPxTG-1, LPxTG-6 and LPxTG-7 (*C*) LPxTG-3, LPxTG-4 and LPxTG-5.

The 33 kb plasmid was named pLPX because it contained a 4 kb gene coding for a surface protein with the cell-wall anchoring LPxTG motif (fig. 5*C*; supplementary table S6, Supplementary Material online). The gene for the LPxTG-containing protein was located next to a gene for a serine recombinase involved in site-specific DNA inversions, suggesting that its expression may be controlled by phase variation. The gene coding for the protein with the LPxTG motif was homologous to more than ten chromosomal genes in the *A. kunkeei* population coding for proteins of variable sizes, of which four to six different gene variants were present in each genome (fig. 6*A*). Different proteins containing the LPxTG motif were located at the same chromosomal site in different strains, indicative of replacement events within and across phylogroups (fig. 6*B*and*C*).

**Fig. 7.**
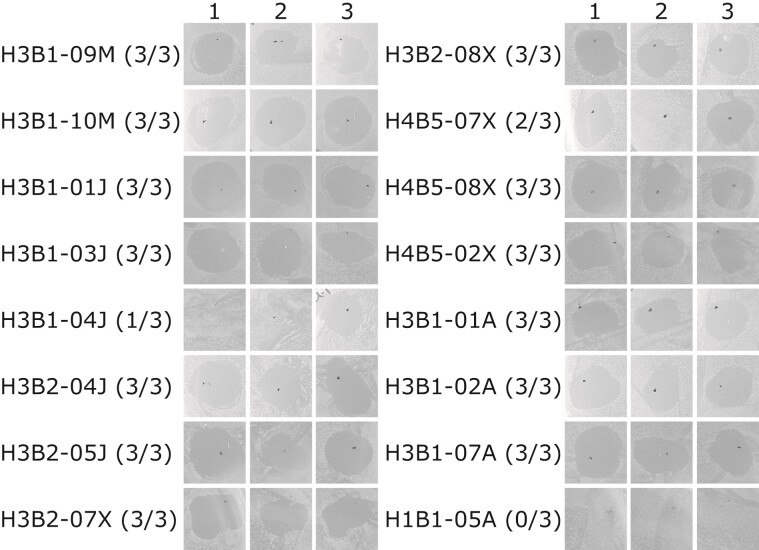
Spot-on-lawn assay for assessing inhibition of *M. plutonius* by *A. kunkeei*. Cell-free supernatants of the *A. kunkeei* strains with predicted pKUN plasmids were added on DSM1582 agar plates with pre-streaked lawns of *M. plutonius*. The figure shows the presence or absence of inhibition zones for the three biological replicates from each strain. The number in parenthesis indicates how many of the biological replicates demonstrated inhibitory potency against *M. plutonius*. Raw images are shown in supplementary figure S6, Supplementary Material online and the data is summarized in supplementary table S7, Supplementary Material online.

Next, we compared the presence of plasmids with the ability of the strains to inhibit growth of *M. plutonius*. For this analysis, we selected a set of 47 strains that represent the genetic diversity of all isolates (see fig. 4), including 31 strains that did not contain the pKUN plasmid and another 16 isolates that contained the plasmid. Fifteen of the 16 isolates that contained the pKUN plasmid showed growth inhibition of *M. plutonius* (fig. 7; supplementary fig. S6 and supplementary table S7, Supplementary Material online). Strain H1B1-05A was the sole isolate that contained the pKUN plasmid but was unable to inhibit growth of *M. plutonius*. Notably, this was also the only isolate in which one gene in the biosynthetic gene cluster was truncated. None of the *A. kunkeei* reference strains without the pKUN plasmid were able to inhibit growth of *M. plutonius* (supplementary fig. S7 and supplementary table S7, Supplementary Material online). Based on these results, we conclude that the plasmid-encoded biosynthetic gene cluster is responsible for growth inhibition of *M. plutonius*.

**Fig. 8.**
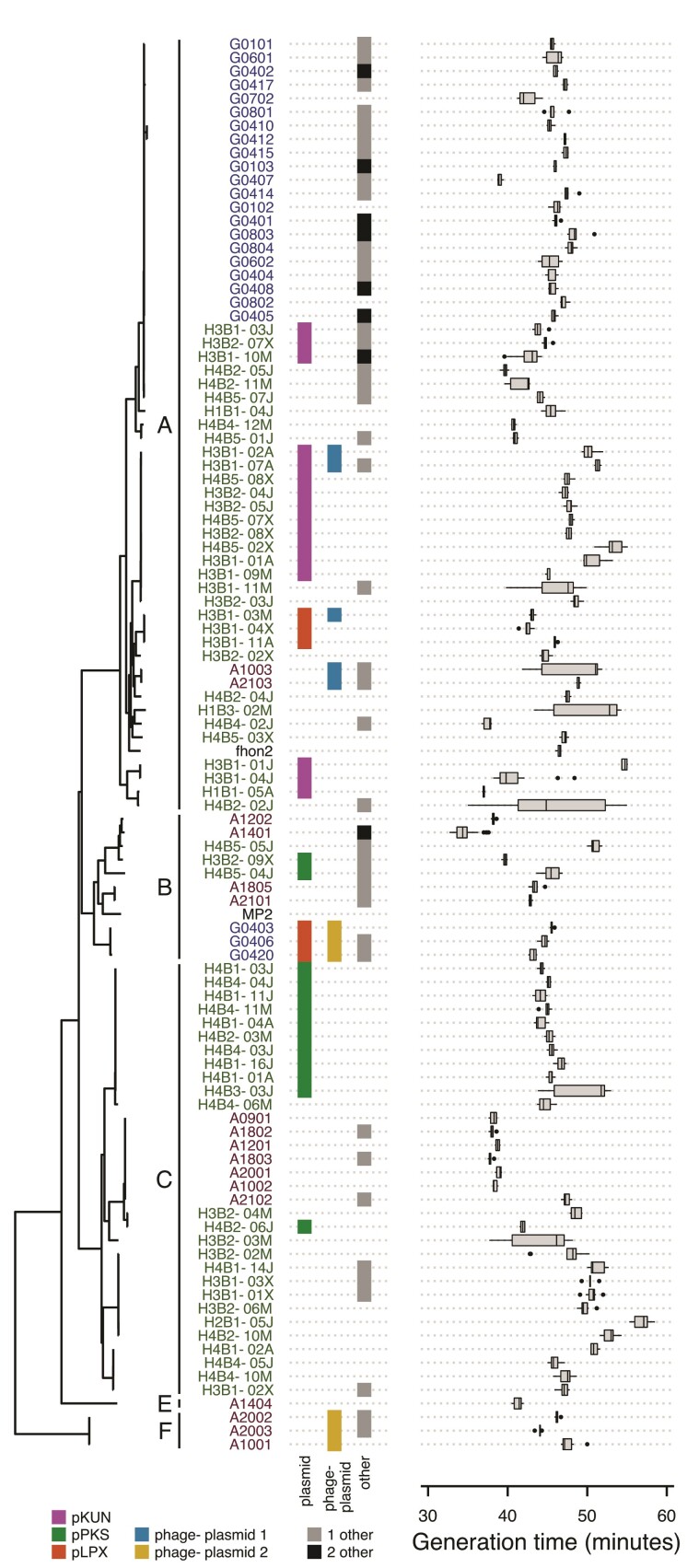
Phyletic distribution patterns of plasmids and phage–plasmids in comparison to generation times. Detailed information about the gene content of plasmids and phage–plasmids is shown in figure 5 and supplementary table S6, Supplementary Material online. Generation times are based on data from multiple experiments per isolate as presented in supplementary table S2, Supplementary Material online. One strain marked with an asterisk had multiple isolates sequenced.

Finally, we examined the phyletic distribution patterns of the extrachromosomal replicons (fig. 8; supplementary table S6, Supplementary Material online). The pKUN plasmid was solely identified in phylogroup A strains from Helsingborg, 13 of 16 of which was sampled from hive 3 (fig. 8; supplementary table S6, Supplementary Material online). The pPKS plasmid was solely identified in phylogroup B and C strains from Helsingborg, 12 of 13 of which were sampled from hive 4. Likewise, the distribution pattern of the two phage–plasmids was phylogroup-specific: one was found in isolates of phylogroup A, while the other was identified in isolates from phylogroups BC and E. Additionally, three phylogroup A isolates from Helsingborg and three phylogroup B isolates from Gotland contained the pLPX plasmid for surface attachment. In contrast to these distinct distribution patterns, the smaller 7–10 kb replicons were broadly present in isolates from all phylogroups and beehives. Generation times were highly variable across isolates irrespectively of their mobile element repertoires (fig. 8; supplementary tables S2 and S3, Supplementary Material online).

No PCR tests were performed to test for infections in the hives, but all bees from which bacterial samples were obtained were healthy at the time of sampling. The identification of antibiotic-producing *A. kunkeei* isolates in samples obtained from the two beehives in Helsingborg may indicate previous exposure to *M. plutonius* or other pathogens, in contrast to the lack of such isolates from the mite-free and mite-resistant bees at Åland and Gotland, respectively.

## Discussion

Insects use bacterial symbionts for the supply of nutrients that are lacking in their diet or as antimicrobial defense systems to tackle the threats from infectious disease agents. There is much hope that understanding the ecology and evolution of the honeybee gut microbiome will help prevent the dramatic losses of managed honeybee colonies in recent years. Due to its identification in the honeycrop and food products in the beehive, *A. kunkeei* is considered to be a core component of the beehive and has been suggested to defend the bees against microbial pathogens. In this paper, we compared the genomes of 104 *A. kunkeei* isolates, including 102 novel genomes and their associated mobilomes. Growth inhibitory effects of the larvae-infecting bee pathogen *M. plutonius* were observed for 15 of these isolates, all of which contained a plasmid for the synthesis of kunkecin A. We also identified a novel plasmid coding for enzymes putatively involved in the synthesis of a polyketide antibiotic. As such, the results strongly suggest the hypothesis that *A. kunkeei* is a defensive symbiont of honeybees.

The comparison of isolates from four different beehives showed that each hive contained a unique composition of strains. Importantly, the same phylogroup strains and type of plasmids were repeatedly sampled from each of the two beehives in Helsingborg during the summer months, despite a continuous turnover of bees and bacterial cells in the hive. This suggests that *A. kunkeei*, like the stable members of the honeybee gut microbiome (Powell et al. 2014; Kwong and Moran 2016), is mainly transmitted through contacts within the hive. Likewise, antibiotic-producing Actinobacteria associated with fungus-farming ant lineages are vertically transmitted across ant colony generations (Li et al. 2018a, 2018b). In contrast, environmental symbionts like *Burkholderia* gut symbionts of stinkbugs are obtained from the environmental microbial pool for every generation (Kikuchi et al. 2011).

Nevertheless, previous studies have shown that *A. kunkeei* strains do not co-speciate with their hosts, suggesting that horizontal transmission of bacteria occurs across sites and host species in the long-term (Tamarit et al. 2015). Likewise, the long-term evolutionary history of the ant-Actinobacteria symbiosis includes horizontal transmission, multiple losses of symbionts, and convergent anatomical adaptations to support the interactions of hosts and symbionts (Li et al. 2018a, 2018b). Thus, although closely related defensive symbionts may show co-divergence with their hosts due to vertical transmission, co-divergence is typically not seen for distantly related organisms due to losses and sporadic horizontal acquisition events (Vorburger and Perlman 2018).

Chemical warfare is a common mechanism whereby symbionts protect their hosts from infections, and the plasmid-encoded molecular defense systems are likely to be adaptive for both *A. kunkeei* and the honeybees. The pKUN plasmid for kunkecin A biosynthesis was mostly associated with phylogroup A strains from Beehive 3 in Helsingborg, while the pPKS plasmid was mostly associated with phylogroups B and C strains from Beehive 4 in Helsingborg. None of the plasmids for antibiotic synthesis were identified in samples from either Gotland or Åland. It is thus likely that selection for plasmids involved in the defense against bee pathogens has driven strains associated with phylogroups A, B and C to high abundances in the beehives located at Helsingborg, while the more diverse bacterial community sampled from the beehive at Åland may have evolved under no or less selection for host-resistance against said pathogen.

The role of geography and phylogeny for horizontal gene transfer and how conditions of rampant gene exchange affect bacterial speciation processes are issues of much debate (Arevalo et al. 2019; Greenlon et al. 2019; VanInsberghe et al. 2020). Local emergence of infectious disease agents may generate geographic structures in defensive symbiont populations as observed for *A. kunkeei*, although most genotypes may be present at most sites. Indeed, selective sweeps driven by antibiotic-producing plasmids may explain the overall abundance of phylogroup A and C strains, as well as the geographic distribution pattern of bacterial isolates and plasmids. Although all bees were healthy at the time of sampling, it is tempting to speculate that the beehives at Helsingborg may have been exposed to bacterial pathogens more recently than the beehives at Åland or Gotland. We do not know if the measures taken to limit the spread of mite infections at Åland have also limited the spread of bacterial pathogens, but it is intriguing to note that the *A. kunkeei* isolates from this island were genetically more diverse than isolates from the other beehives. In the long-term, host-selected expansion of microbial defense systems could lead to segregation of populations into distinct gene-flow units and thereby drive niche-specialization and speciation. *Apilactobacillus kunkeei* may thus be used as a model system for studies of niche-specialization and speciation processes in defensive symbiont populations.

It is also of interest to note that *A. kunkeei* circulates among several microhabitats in the beehive and growth of the bacterial population may not necessarily occur in the same habitat as antibiotic synthesis. Such a separation of growth from antibiotic synthesis has been observed for example in the defensive symbiont of beewolf digger wasps, which grows in the antennae of adult female wasps but produces antibiotics in the brood cells and larvae (Kaltenpoh et al. 2010; Kroiss et al. 2010). Furthermore, some polyketides, such as actinorhodin produced by *Streptomyces coelicolor* (Wright and Hopwood 1976) are synthesized under oxygen-rich conditions at high pH while the compound itself is most active at low pH (Mak and Nodwell 2017). It is thus tempting to speculate that the antibiotic synthesized by enzymes encoded by the pPKS plasmid may be most active in microhabitats of low pH, such as in the fresh honey and royal jelly. Restricting the activity of the antimicrobial compounds to the honeybee food products may be a mechanism to target pathogens that infect the larval food without harming other members of the honeybee microbiome, provided that the antibiotics are not stable enough to persist in the bee food.

*Bombella apis* is also found in multiple and similar microhabitats within the hive, such as the nurse crop, nectar, larval diet and royal jelly. It is shown that *B. apis* can synthetize all essential amino acids and secrete lysine, suggesting that this bacterial species may serve as a nutritional symbiont of honeybee larvae (Parish et al. 2022). Thus, *B. apis* and *A. kunkeei* may jointly support larval development by complementing the larval diet and protecting it against infections. Social insects provide a model for how the transmission of infectious disease agents may be reduced in animal communities with high population densities by incorporating beneficial microbes into the host–parasite co-evolutionary arms race. At the more advanced stages of these interactions, the defensive symbionts may take over the interactions with the pathogens, thereby relaxing selection on the host-derived immune system.

Taken together, the results presented in this study suggest that the *A. kunkeei* community serves an important role in bee health by protecting the larvae and their diet against infections. Future studies should be targeted towards characterizing the putative antibiotics, the microhabitat in which they are synthesized and their mode of action on known bee pathogens. We have currently no evidence to suggest that the honeybees regulate the mobilome and other activities of *A. kunkeei*, nor that the adoption of defensive symbionts has had an impact on the honeybees own immune system against infectious disease agents, but this is an interesting avenue for further studies. Alternatively, selection may simply have favored bees containing the best fitted *A. kunkeii* strains. The availability of more than 100 *A. kunkeei* isolates with different gene complements and plasmids now enable studies to address the long-standing debate about whether horizontally transferred genes are mostly neutral, deleterious or adaptive (discussed in Rocha 2018). Importantly, the results suggest that the complex interactions of hosts, defensive symbionts and pathogens cannot be adequately understood unless the dynamics of the mobile gene pool of the symbionts are incorporated into the models. Understanding these interactions will be important for the design of engineered strains and plasmids to improve the health of honeybees.

## Materials and Methods

### Sample Collection, Bacterial Cultivation and Inhibition Studies

Honeybees from the subspecies *Apis mellifera* were collected from beehives located on the islands Åland and Gotland as well as near Helsingborg, Honeybees from Åland and Gotland were stored at −80 °C. Prior to dissection, honeybees were thawed and placed individually in 2 ml ethanol. Honeybees from Helsingborg were dissected immediately after collection as described in (Olofsson and Vasquez 2008; Vasquez and Olofsson 2009). The honeycrops were extracted and homogenized in PBS and spread on MRS agar (Sigma Aldrich), supplemented with 0.5% fructose and 1.5% glycine (samples from Åland and Gotland) or 2% fructose 0.1% cysteine (samples from Helsingborg). Bacterial isolates were obtained after incubation at 35 °C and 5% CO_2_ for 2–3 days and re-isolated to obtain pure isolates in the same growth media. The isolates were examined by PCR and Sanger sequencing using universal 16S rRNA primers and MALDI-TOFMS protein profiling as described in (Olofsson et al. 2014).

For estimates of generation times, the *A. kunkeei* strains were first grown on MRS agar plates supplemented with 0.5% D-Fructose (fMRS agar) overnight at 35 °C, 5% CO_2_. To obtain biological replicates, several single colonies were picked per strain to inoculate liquid MRS medium (+0.5% D-Fructose, fMRS medium) and two rounds of pre-cultures were incubated for 18 h at 35 °C, 5% CO_2_ after which the concentration of the cell suspensions was adjusted to OD600 = 0.005 in fresh fMRS in multi-well plates and the absorbance at λ = 600 nm was monitored in 10 min intervals for up to 24 h on a Bioscreen C MBR at a temperature of 35 °C (Oy Growth Curves Ab Ltd). Cultures were kept on ice during sample preparation. Biological triplicates of *A. kunkeei* strain A1401 were included as a positive control in every experiment. Non-inoculated fMRS medium served as negative control. Raw data was log-transformed, background corrected, trimmed after 10 h and the growth dynamics were determined in R (R Core Team) using the growthcurver package (Sprouffske and Wagner 2016).

To test the inhibitory potency of *A. kunkeei* against *M. plutonius* (DSM29964), cell-free supernatants were isolated from 47 *A. kunkeei* strains. The strain collection included 33 representative strains shown in figure 4*B*, excluding *A. kunkeei* MP2, and, in addition, 14 *A. kunkeei* strains with predicted pKUN plasmids. *Apilactobacillus kunkeei* strains H1B1-05A and H3B1-04J with predicted pKUN plasmids were part of the representative strain collection. To obtain the cell-free supernatant, the bacterial cells were cultivated as described for the growth analysis. After the final batch cultivation in fMRS medium for 18 h at 35 °C, 5% CO, cells were pelleted by centrifugation (4,500 × g, 10 min, 4 °C) and the supernatant was passed through 0.2 µm membrane filters. *Melissococcus plutonius* (DSM29964) was cultivated in DSM 1582 medium at 30 °C for 3 days under anaerobic conditions. Anaerobic growth conditions were created in an Anaerocult jar (Millipore) using Anaerocult A bags and monitored using Anaerotest Strips. For the inhibition studies, *M. plutonius* was diluted to a cell concentration corresponding to OD600 of 0.05–0.2 and evenly spread on DSM 1582 agar plates. In a spot-on-lawn assay, 10 µl of *A. kunkeei* cell-free supernatants were added upon the *M. plutonius* cell lawn and plates were incubated anaerobically at 30 °C for at least 2 days. Inhibition was assessed if a clear inhibition zone was observed after cultivation. Experiments were performed with biological triplicates, cell-free supernatants from strains H3B1-09M and H3B1-10M were used as positive control and fMRS medium was included as a negative control.

### Genome Sequencing, Assembly, and Annotation

The genomes of the 102 novel *A. kunkeei* isolates as well as the genomes of the previously isolated *A. kunkeei* Fhon2 and *A. apinorum* Fhon13 strains were sequenced with PacBio RS II and PacBio Sequel technologies. The reads from each sequencing run were assembled into closed genomes with HGAP3 or HGAP4 (Chin et al. 2013), as detailed in (supplementary table S1, Supplementary Material online). MUMmer v3.23 (Kurtz et al. 2004) was used to rule out mis-assemblies. Pairwise ANI values between chromosomes were calculated using FastANI v1.2 with default parameters.

The manually annotated genome of *A. kunkeei* Fhon2 (Tamarit et al. 2015) was used as reference by the Prokka v1.14.6 annotation pipeline (Seemann 2014) and complemented by searches against all bacterial sequences in UniProtKB (The UniProt Consortium 2020). The eggNOG-mapper v2.1.2 (Huerta-Cepas et al. 2016) and InterProScan v5.51-85.0 (Jones et al. 2014) were applied to the genomes using default parameters. All genomes were scanned for transposable elements, deduced from prokka, eggNOG, and InterProScan functional predictions. Proteins were sorted into Clusters of Orthologous Groups (COGs) using BLASTP against the COG2020 database (Galperin et al. 2021) and discarding overlapping hits. Prophages were predicted using PHASTER with Prophage/Virus database as of December 22, 2020 (Arndt et al. 2016).

Extrachromosomal elements were assembled with Flye v2.8.3-b1725 (Kolmogorov et al. 2019) with the –plasmid parameter and –asm_coverage set to 100, 150, 200, and 500. The resulting non-chromosomal contigs were grouped based on a combination of shared gene content, reciprocal blast hits, and ANI values. For each strain containing elements assigned to a given group, the contig with the length closest to the median length of that group was selected as the reference assembly. The two phage–plasmids were classified by taking protein sequences from one representative each and using protein BLAST against all phages described in (Pfeifer et al. 2021) and counting hits. Plasmids and phage–plasmids of 20 kb or more were ordered using the *repA*, *parE*, and *repB* genes. Extrachromosomal contigs solely present in a single strain often contained transposons and homologs to chromosomal genes. These elements were not further examined in this study, except for the transposon-containing contig identified in the assembly of isolate H3B2-03M.

### Phylogenetic Analyses

The previously published closed genome of *A. kunkeei* strain MP2 (Asenjo et al. 2016) and the non-closed genomes of 15 *A. kunkeei* strains (Djukic et al. 2015; Porcellato et al. 2015; Sun et al. 2015; Tamarit et al. 2015) (supplementary table S8, Supplementary Material online) were re-annotated using the Prokka v1.14.6 annotation pipeline (Seemann 2014) for consistency with the 102 novel genomes. The inferred proteomes of the 102 novel *A. kunkeei* genomes, the *A. kunkeei* Fhon2 and *A. apinorum* Fhon13 genomes, and the 16 previously sequenced *A. kunkeei* genomes were sorted into protein families using OrthoMCL v2.0 (Li et al. 2003).

For the phylogenetic analysis, we selected a subset of core protein families present in all *A. kunkeei* strains and *A. apinorum* Fhon13 after exclusion of protein families with multiple members in any single genome, proteins shorter than 100 amino acids, and proteins inferred to be recombinant by all tests in the software PhiPack v1.1 (Bruen et al. 2006). The selected proteins were individually aligned with mafft-linsi v7.453 (Katoh et al. 2002) allowing regions with gaps (option –leavegappyregion). Sequences were trimmed using trimal v1.4rev15 (Capella-Gutiérrez et al. 2009) with automatic detection of optimal thresholds (-gappyout) and concatenated. A phylogenetic tree was inferred with IQ-Tree v1.6.10 (Nguyen et al. 2015), using the LG + F + R5 substitution model, selected using ModelFinder (Kalyaanamoorthy et al. 2017). A total of 1,000 ultrafast bootstrap (option *-bb 1000*) and SH-like (option *-alrt 1000*) pseudoreplicates were performed to assess branch support and stability. *Apilactobacillus apinorum* strain Fhon13 was used to root the tree.

For the pan-genome analysis, we selected protein families containing proteins encoded by chromosomal genes in at least one of the 104 *A. kunkeei* isolates with closed genomes. The protein families were classified as core, soft-core, shell, and cloud depending on whether they contained proteins in all 104 isolates (100%), ≥99 (95%), ≥16 (15%), or ≤15 isolates, respectively.

## Supplementary Material

evac153_Supplementary_Data

## Data Availability

The *A. kunkeei* genome sequences have been deposited in the European Nucleotide Archive under accession numbers GCA_946887455-GCA_946888655 (supplementary table S1, Supplementary Material online). Genome assembly statistics for the genomes sequenced as part of this study along with gene predictions for the previously published genomes, protein families, protein alignments, inhibition assays, and all underlying code used for this project are available at the SciLifeLab Data Repository under https://doi.org/10.17044/scilifelab.c.6174535.
