# Supplementary material for: Genome Evolution of a Symbiont Population for Pathogen Defense in Honeybees
Source: Genome Biol Evol. 2022 Oct 20;14(11):evac153. doi: 10.1093/gbe/evac153 (PMC9648514; doi:10.1093/gbe/evac153)
Supplement: evac153_Supplementary_Data [file evac153_supplementary_data.zip › Suppl.Figures.pdf]

SUPPLEMENTARY INFORMATION

Genome evolution of a symbiont population for pathogen defence in honeybees

Karl Dyrhage[1*], Andrea Garcia-Montaner[1*], Daniel Tamarit[1], Christian Seeger[1], Kristina Näslund[1], Tobias C. Olofsson[2], Alejandra Vasquez[2], Matthew T. Webster[3], Siv G. E. Andersson[1]

SUPPLEMENTARY FIGURE LEGENDS

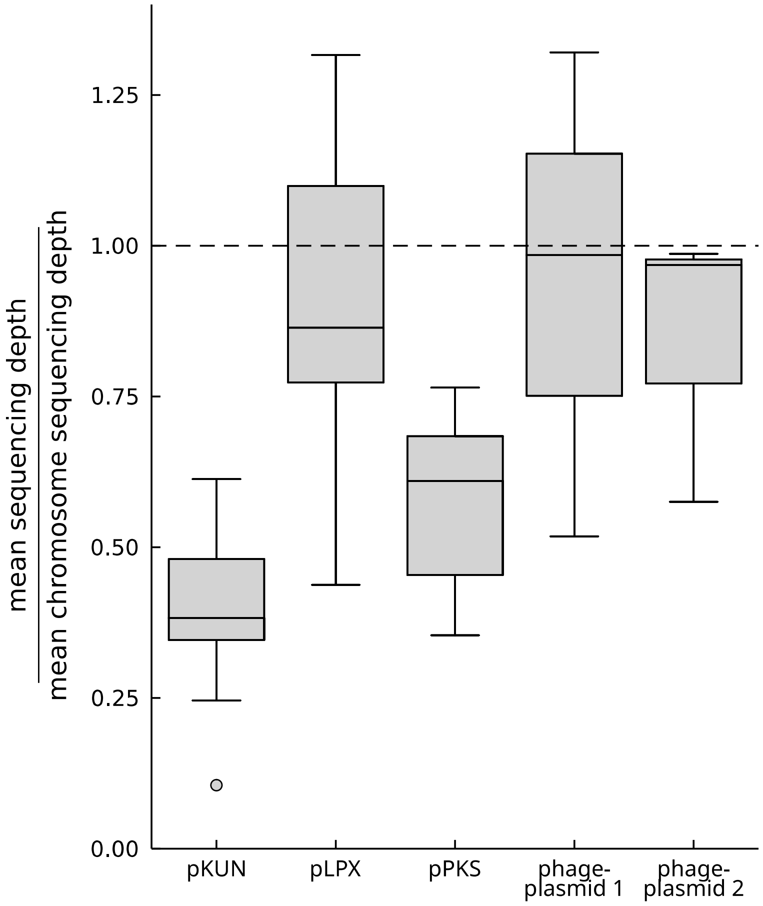

Figure S1. Read map depth over plasmids and phage-plasmids. The depth of sequence reads on the plasmids and phage-plasmids compared to the chromosome averaged per type of element.

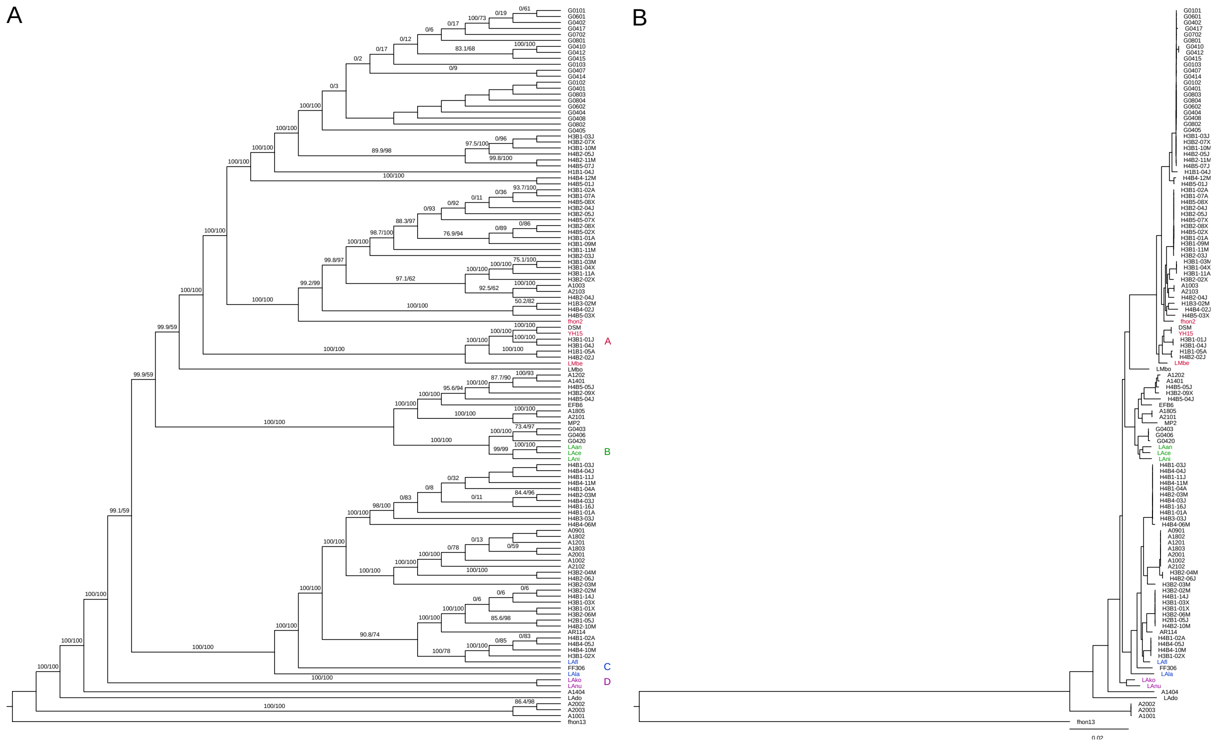

Figure S2. Phylogenetic representation of *A. kunkeei* isolates. The tree is displayed **A** in a schematic form (cladogram) and **B** such that branch lengths are proportional to substitution frequencies. The tree displayed in **A** shows the percentage support values for each node obtained from 1000 ultrafast bootstrap and SH-like pseudoreplicates. The previously sequenced *A. kunkeei* strains with phylogroup designations (Tamarit et al. 2015) are coloured and marked with letters A-D. The phylogeny was obtained using IQ-Tree with the LG+F+R5 model based on 682 single-copy orthologous proteins in the 102 closed *A. kunkeei* genomes of this study as well as the 16 previously sequenced *A. kunkeei* genomes (Djukic et al. 2015; Sun et al. 2015; Tamarit et al. 2015; Asenjo et al. 2016) along with the re-sequenced and closed *A. kunkeei* Fhon2 and *A. apinorum* Fhon13 genomes. The root was placed on the branch to *A. apinorum* Fhon13.

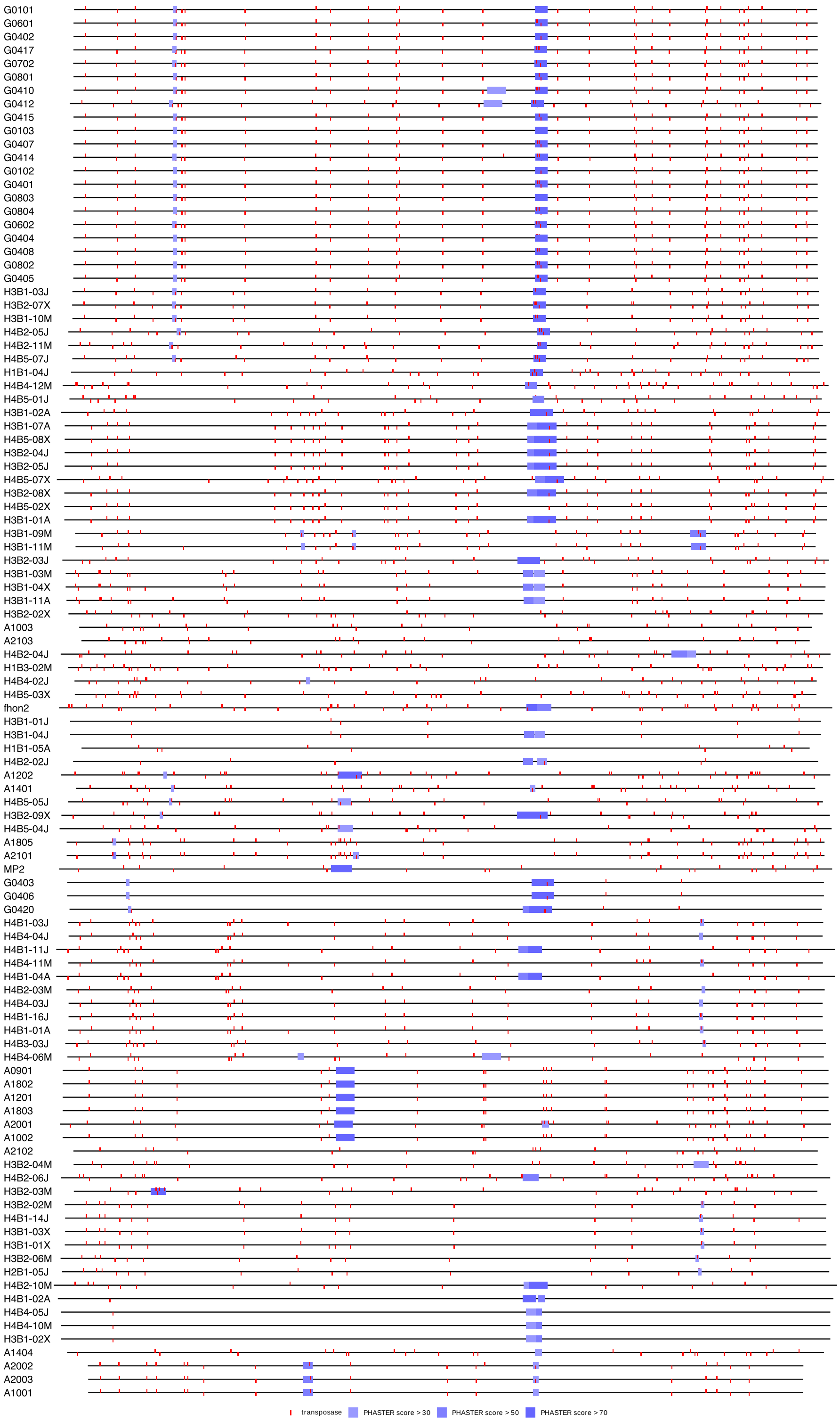

Figure S3. Locations of prophages and transposons in the *A. kunkeei* genomes. The location of prophages and transposons in genomes from all isolates, color-coded according to phylogroups. Vertical red lines above or below chromosome lines indicate transposase genes located in the Watson or Crick strands, respectively. Blue boxes on the chromosomes represent prophage genes predicted by PHASTER (score more than 30). Gray, connecting lines represent Blastn hits with e-values lower than 1e-5.

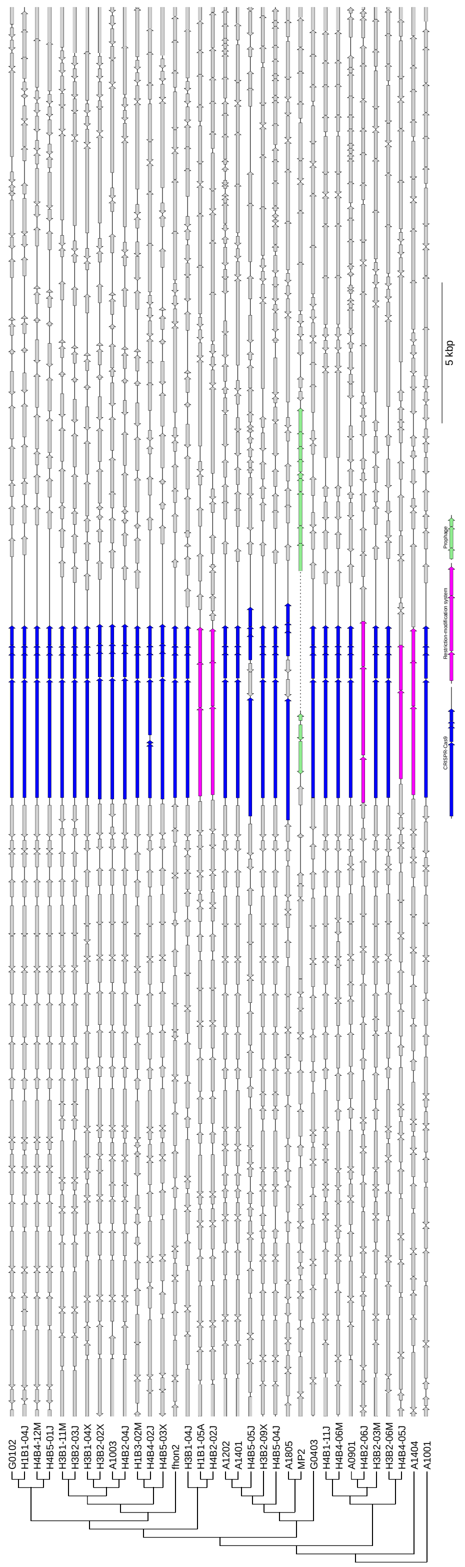

Figure S4. **Phage defence genomic island.** The plot shows a chromosomal region that encodes phage defence systems, either CRISPR-Cas (blue) or restriction-modification (magenta) system. One strain has a prophage (green) which has been truncated to fit in the figure. Sequence similarity between genome regions is represented by Blastn hits with an e-value of 1e-10 or lower.

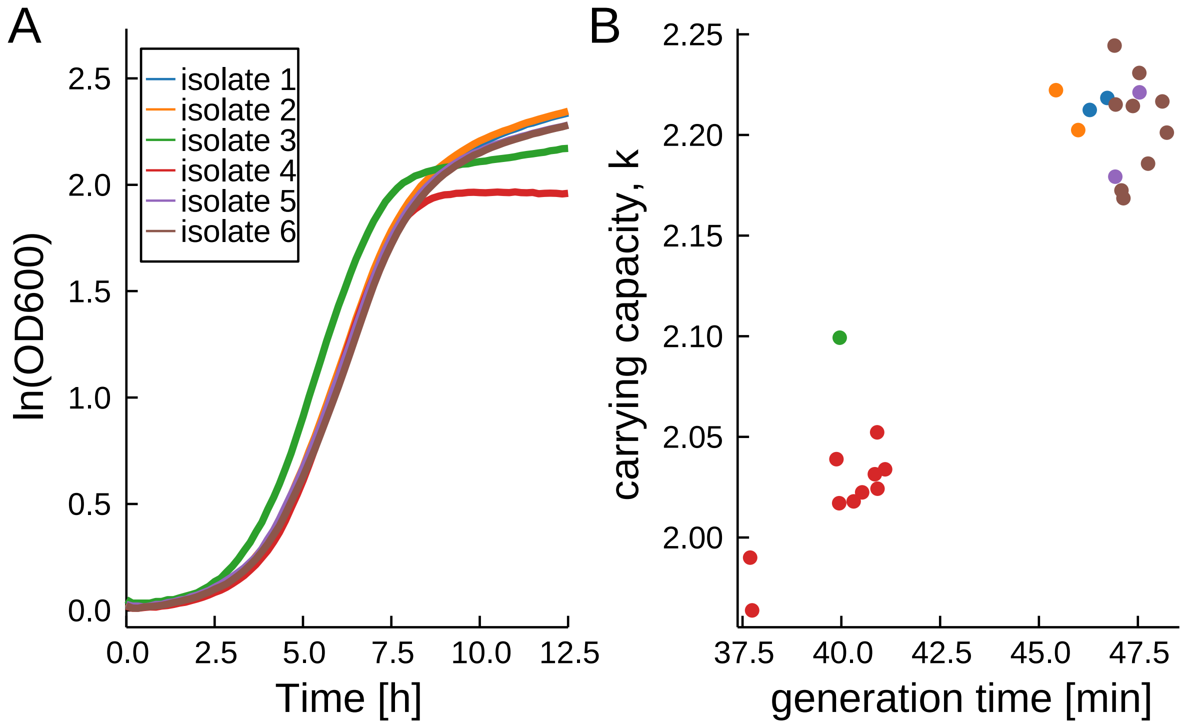

Figure S5. Growth analysis of H3B2-03M colonies. Background-corrected and log-transformed OD600-values for the different H3B2-03M colonies are plotted in **A** as a function of the measurement time. In **B**, the estimated carrying capacity (k) of each replicate of the respective H3B2-03M isolates is plotted as a function of the estimated generation time.

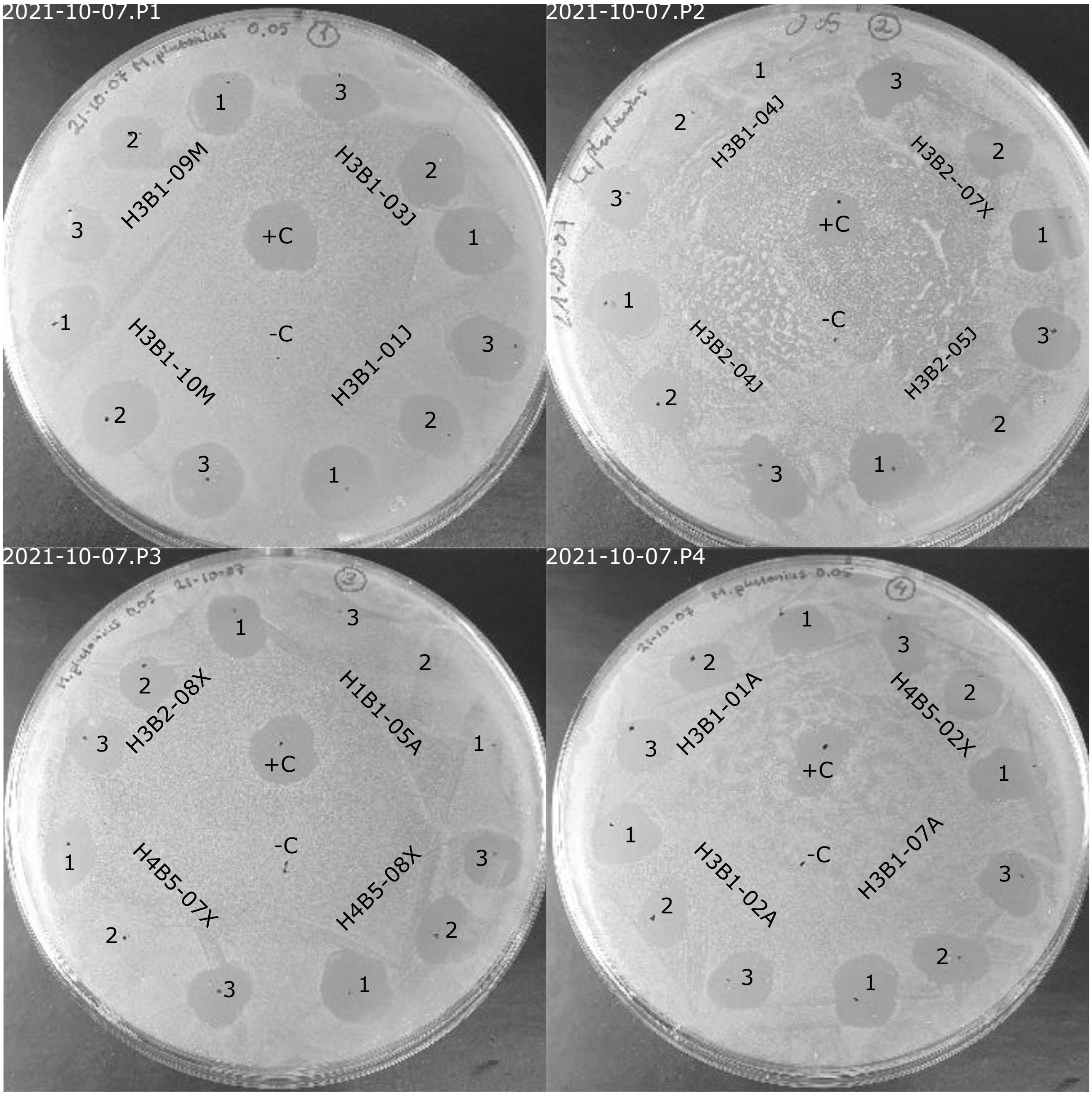

Figure S6. Spot-on lawn inhibition assay against *M. plutonius*. Cell-free supernatants of the *A. kunkeei* strains with predicted pKUN plasmids were added on DSM 1582 agar plates with pre-streaked lawns of *M. plutonius*. The strain names are indicated next to the spots where the biological replicate samples (1–3) have been added. Positive (“+C”, cell-free supernatants from H3B1-09M) and negative controls (“-C”, fMRS medium) were added in the middle of the plates.

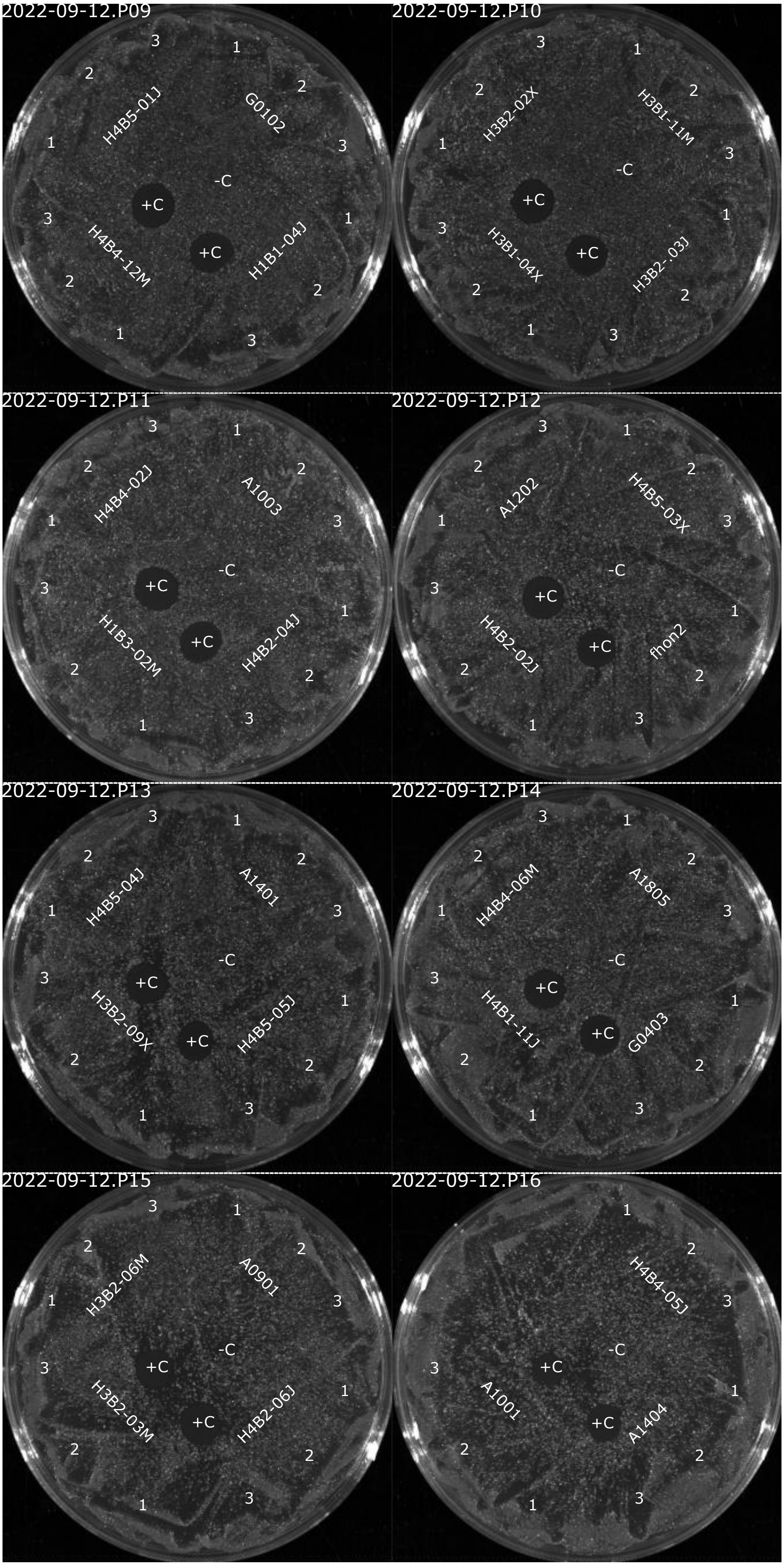
2022-09-12.P09
H4B5-01J
G0102
-C
+C
+C
H4B4-12M
H1B1-04J
2022-09-12.P10
H3B2-02X
H3B1-11M
-C
+C
+C
H3B1-04X
H3B2-.03J
2022-09-12.P11
H4B4-02J
A1003
-C
+C
+C
H1B3-02M
H4B2-04J
2022-09-12.P12
A1202
H4B5-03X
-C
+C
+C
H4B2-02J
fhon2
2022-09-12.P13
H4B5-04J
A1401
-C
+C
+C
H3B2-09X
H4B5-05J
2022-09-12.P14
H4B4-06M
A1805
-C
+C
+C
H4B1-11J
G0403
2022-09-12.P15
H3B2-06M
A0901
-C
+C
+C
H3B2-03M
H4B2-06J
2022-09-12.P16
H4B4-05J
-C
+C
+C
A1001
A1404

Figure S7. Spot-on lawn inhibition assay against *M. plutonius*. Cell-free supernatants of the *A. kunkeei* strains without predicted pKUN plasmids were added on DSM 1582 agar plates with pre-streaked lawns of *M. plutonius*. The strain names are indicated next to the spots where the biological replicate samples (1–3) have been added. Positive (“+C”, cell-free supernatants from H3B1-09M and H3B1-10M) and negative controls (“-C”, fMRS medium) were added in the middle of the plates.

SUPPLEMENTARY TABLE LEGENDS

Table S1. Summary of all *A. kunkeei* isolates including the site and beehive they were obtained from and the sampling time.

Table S2. Growth rate estimates for the *A. kunkeei* isolates using a logistic growth model.

Table S3. Genome information of the novel 102 *A. kunkeei* isolates, the re-sequenced *A. kunkeei* Fhon2 and *A. apinorum* Fhon13 strains as well as the re-sequenced *A. kunkeei* isolate H3B2-03M that segregated into colonies with different growth kinetics. For each isolate, the 16S rRNA similarities against Fhon2 and Fhon13 are shown for the least and most divergent 16S rRNA gene copies.

Table S4. Protein families including proteins from *A. kunkeei* strains with complete genomes. The table includes information about gene IDs, gene lengths, recombinant genes, COG classifications, pangenome classifications, and the protein families that were used for the phylogenomic inference. The protein families have been extracted from a clustering performed with OrthoMCL for the full set of 118 closed and non-closed *A. kunkeei* genomes.

Table S5. Average nucleotide sequence identity values. Pairwise average nucleotide sequence identities (ANI) of the *A. kunkeei* strains.

Table S6. Annotations of genes located on plasmids and phage-plasmids.

Table S7. Inhibition of *M. plutonius* by *A. kunkeei* strains. Strains, predicted plasmids, classification as representative strains are indicated. Inhibition experiments were performed with biological triplicate samples on the indicated dates and respective plates. Strains were regarded as positive for inhibition of *M. plutonius* when they showed inhibition in at least one of three replicates.

Table S8. Summary of previously published *A. kunkeei* genomes used in the study.
